# Lung ultrasound for the diagnosis of pneumonia in children with acute bronchiolitis

**DOI:** 10.1186/s12890-018-0750-1

**Published:** 2018-12-07

**Authors:** Carlotta Biagi, Luca Pierantoni, Michelangelo Baldazzi, Laura Greco, Ada Dormi, Arianna Dondi, Giacomo Faldella, Marcello Lanari

**Affiliations:** 1 Pediatric Emergency Unit, Department of Medical and Surgical Sciences (DIMEC), St. Orsola-Malpighi Hospital, University of Bologna, Via Massarenti 11, 40138 Bologna, Italy; 2 Pediatric Radiology Unit, S.Orsola-Malpighi Hospital, University of Bologna, Bologna, Italy; 3 Department of Medical and Surgical Sciences (DIMEC), University of Bologna, Bologna, Italy; 4 Neonatology and Neonatal Intensive Care Unit, Department of Medical and Surgical Sciences (DIMEC), St. Orsola-Malpighi Hospital, University of Bologna, Bologna, Italy

**Keywords:** Lung ultrasound, Pneumonia, Bronchiolitis, Chest X-ray

## Abstract

**Background:**

Guidelines currently do not recommend the routine use of chest x-ray (CXR) in bronchiolitis. However, CXR is still performed in a high percentage of cases, mainly to diagnose or rule out pneumonia. The inappropriate use of CXR results in children exposure to ionizing radiations and increased medical costs. Lung Ultrasound (LUS) has become an emerging diagnostic tool for diagnosing pneumonia in the last decades. The purpose of this study was to assess the diagnostic accuracy and reliability of LUS for the detection of pneumonia in hospitalized children with bronchiolitis and to evaluate the agreement between LUS and CXR in diagnosing pneumonia in these patients.

**Methods:**

We enrolled children admitted to our hospital in 2016–2017 with a diagnosis of bronchiolitis and undergone CXR because of clinical suspicion of concomitant pneumonia. LUS was performed in each child by a pediatrician blinded to the patient’s clinical, laboratory and CXR findings. An exploratory analysis was done in the first 30 patients to evaluate the inter-observer agreement between a pediatrician and a radiologist who independently performed LUS. The diagnosis of pneumonia was established by an expert clinician based on the recommendations of the British Thoracic Society guidelines.

**Results:**

Eighty seven children with bronchiolitis were investigated. A final diagnosis of concomitant pneumonia was made in 25 patients. Sensitivity and specificity of LUS for the diagnosis of pneumonia were 100% and 83.9% respectively, with an area under-the-curve of 0.92, while CXR showed a sensitivity of 96% and specificity of 87.1%. When only consolidation > 1 cm was considered consistent with pneumonia, the specificity of LUS increased to 98.4% and the sensitivity decreased to 80.0%, with an area under-the-curve of 0.89. Cohen’s kappa between pediatrician and radiologist sonologists in the first 30 patients showed an almost perfect agreement in diagnosing pneumonia by LUS (K 0.93).

**Conclusions:**

This study shows the good accuracy of LUS in diagnosing pneumonia in children with clinical bronchiolitis. When including only consolidation size > 1 cm, specificity of LUS was higher than CXR, avoiding the need to perform CXR in these patients. Added benefit of LUS included high inter-observer agreement.

**Trial registration:**

Identifier: NCT03280732. Registered 12 September 2017 (retrospectively registered).

## Background

Bronchiolitis is a viral lower respiratory tract infection that affects children younger than 24 months and represents the leading cause of hospitalization in infants [1]. The main responsible pathogen is Respiratory Syncytial Virus (RSV), with infection typically occurring as recurrent seasonal epidemics [2, 3]. The treatment is primarily supportive and no specific etiological therapy is routinely used to limit the viral infection and reduce the severity of clinical course [4, 5].

According to the most recent American Academy of Pediatrics guidelines [4], the diagnosis of bronchiolitis is clinical and chest x-ray (CXR) should be reserved for severe cases in which signs of pulmonary complications are present or where the severity of respiratory effort leads to Intensive Care Unit (ICU) admission.

Nevertheless, there is high variation in use of diagnostic tests across hospitals and CXR is still performed in about 50% of bronchiolitis [6–8], mainly to diagnose or rule out bacterial pneumonia. It has been shown that children with clinical bronchiolitis are more likely to receive antibiotics when radiography is performed owing to similar radiographic appearance of infiltrate and atelectasis [9]. Moreover, even if in clinical practice alveolar infiltration is considered to be secondary to bacterial infection and bilateral interstitial infiltrates to atypical bacterial or viral infections, CXR is too insensitive to distinguish bacterial from viral pneumonia [10]. Finally, radiographic images interpretation varies significantly among observers [11].

Despite these well-known limitations, CXR is widely used in bronchiolitis, resulting in children exposure to ionizing radiations, increased medical costs, time spent, and potential complications due to unnecessary antibiotic prescription [12, 13]. For this reason, in the last years many quality improvement methodologies have been attempted to minimize x-ray use in these patients [14, 15]. Despite these attempts, no significant enhancement into clinical practice has been reached.

Lung Ultrasound (LUS) is a feasible, portable, easy to learn and non ionizing radiation technique. In the last decades it has become an emerging diagnostic tool for diagnosing pneumonia in adults and children, with remarkable sensitivity and specificity [16–20]. Moreover, in the last years there has been great interest in using LUS to differentiate bacterial pneumonia from viral infections [21, 22]. In this sense LUS may be the ultimate tool to diagnose or rule out bacterial pneumonia in children with clinical bronchiolitis and to identify who would benefit from antibiotics. Nevertheless, at present LUS is not included in the diagnostic work-up of bronchiolitis. In fact, although few studies describe the sonographic characteristics of bronchiolitis [23, 24], none have investigated the role of LUS in children with clinical bronchiolitis and suspected pulmonary bacterial co-infection.

To our knowledge this is the first study to investigate the role of LUS in diagnosing pneumonia in children with acute bronchiolitis. The primary aim of this study was to assess the diagnostic accuracy and reliability of LUS for the detection of pneumonia in children with bronchiolitis and to evaluate the agreement between LUS and CXR in diagnosing pneumonia in these patients. Furthermore, we evaluated the interobserver agreement of LUS between a pediatric clinician and a pediatric radiologist who independently performed LUS.

## Methods

This is a prospective study performed at the Pediatric Emergency Unit of S.Orsola-Malpighi Hospital (Bologna, Italy) in association with the Pediatric Radiology Unit during two consecutive autumn and winter seasons (2016–2017). The study was approved by the ethics committee of our institution. Parents of all the eligible patients accepted to participate in the study and gave informed written consent.

### Study population

Inclusion criteria consisted of children from birth to 24 months of age admitted to our hospital from February 2016 to April 2017 with a diagnosis of bronchiolitis according to the American Academy of Pediatrics guideline [4] and undergone posteroanterior CXR because of clinical suspicion of concomitant bacterial pneumonia. Bacterial pneumonia was suspected in patients with at least one of: fever > 38.5 °C or > 38 °C for 2 or more days, persistent oxygen saturation (SatO2) < 92%, asymmetric breath sounds on auscultation, abnormal laboratory investigations - White Blood Cells (WBC) > 15,000/mmc and/or C-Reactive Protein (CRP) > 4 mg/dl - or septic appearance. According with the British Thoracic Society guidelines, lateral radiographs were not routinely performed to avoid unnecessary exposure to further radiation [10]. Exclusion criteria were chronic respiratory disease (i.e. bronchopulmonary dysplasia), congenital heart diseases, severe neuromuscular disease and congenital or acquired immunodeficiency.

The diagnostic gold standard for the study was the ex-post diagnosis of bacterial pneumonia made by an experienced paediatrician blinded to LUS findings, on the basis of clinical presentation, laboratory tests, CXR and clinical course following British Thoracic Society Guidelines recommendations [10].

### LUS examination

All patients underwent a bedside LUS in the first 12 h after CXR. LUS was performed using a Mindray-DC-T6 ultrasound machine equipped with a linear probe with frequencies ranging from 7.5 MHz to 12 MHz. LUS examinations were done according to the methodology described by Copetti and Cattarossi [25]. To cover the whole lung surface, each hemithorax was divided into three areas: the anterior area delimited by parasternal and anterior axillary lines, the lateral area between the anterior and posterior axillary lines, and the posterior area delimited by the paravertebral and posterior axillary lines. Each region was scanned in the longitudinal and transverse plane, medial-lateral and up-down respectively. The anterior and lateral regions of the chest were examined while the infants in supine decubitus. The posterior region was examined in prone decubitus in infants while sitting position was used to scan the posterior wall in older patients.

LUS was performed by a pediatrician with specific LUS expertise and unaware of the clinical, laboratory and radiographic data of the patients. The pediatrician has attended a 8-h LUS training session and supervised practical training.

An exploratory analysis was used in the first 30 patients to evaluate the sonographer inter-observer concordance between the pediatrician and a pediatric radiologist. The radiologist performed the LUS right after the pediatrician, being blinded to the results of the previous LUS and CXR studies. Similarly, two pediatric radiologists independently reviewed the radiographic images of the same set of patients to evaluate the inter-observer concordance of the CXR. The radiological images which were contradictory were re-evaluated by a senior radiologist to reach a final interpretation.

**Fig. 1:**
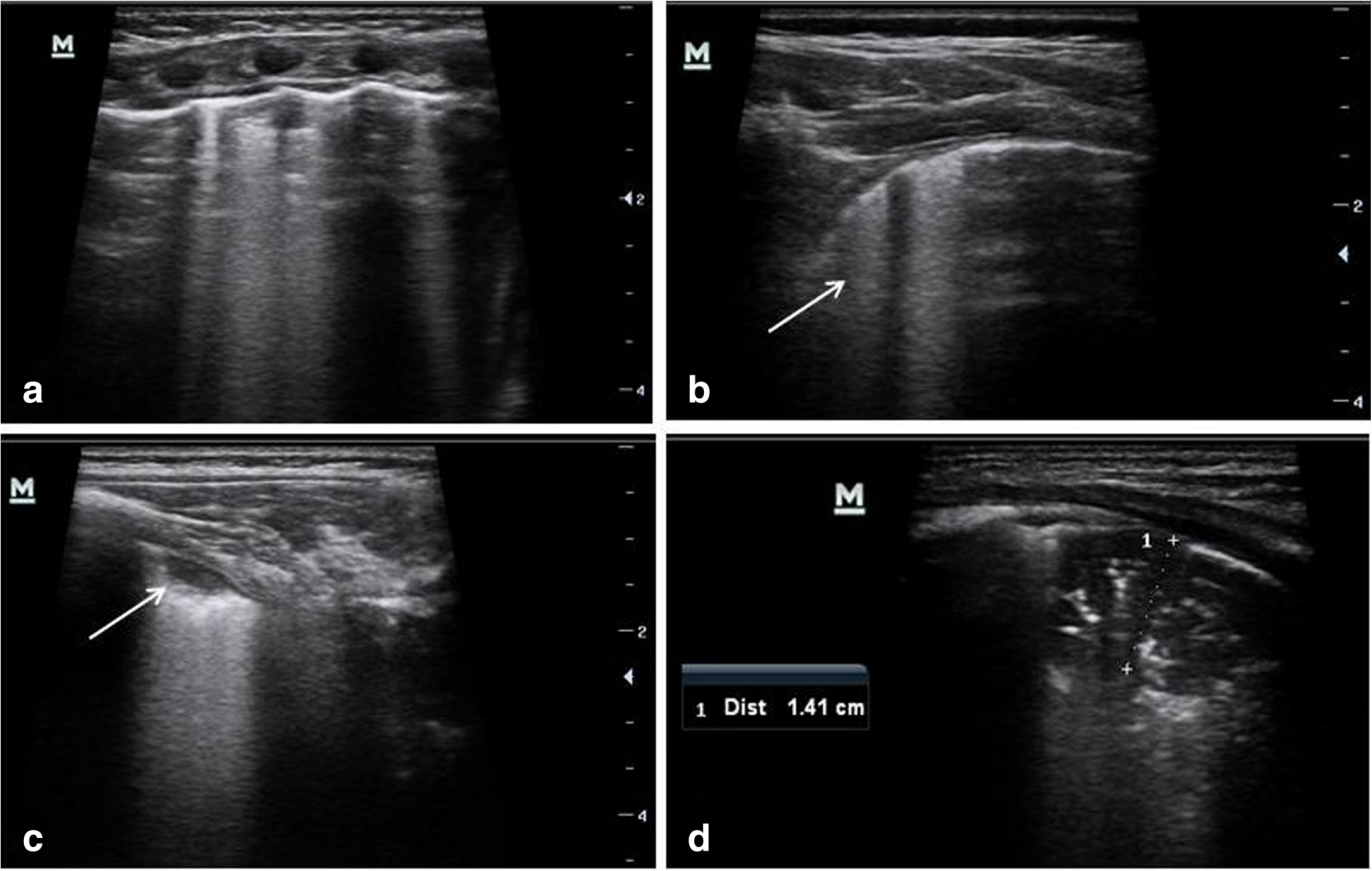
Lung ultrasound images in a patient with bronchiolitis complicated by pneumonia. **a** Transversal intercostals approach showed multiple B lines, consistent with bronchiolitis. **b** Longitudinal thoracic scan, revealed irregular pleural surface and confluent B lines (arrow). **c** The left posterior lung field showed a small subpleural consolidation without sonographic air bronchograms (arrow) - a typical finding in infants with bronchiolitis – associated with focally confluent B lines arising from the margin of the consolidation. **d** The scan of the posterior region of the right lung revealed a consolidation with hyperechoic air bronchograms suggestive of pneumonia

The criterion to define pneumonia on LUS was the finding of an hypoechogenic area with poorly defined borders and compact underlying artifacts perpendicular to the pleural line, called B lines [25]. The pleural line is less echogenic in the area interested by consolidation and lung sliding is reduced or absent. Similar to prior studies [16, 20, 21], bacterial pneumonia was defined as lung consolidation with air bronchograms (Fig. 1). In pneumonia air bronchograms appear in an scattered dot-like and branching pattern. By contrast, in atelectasis, the airless lung is similar in echogenicity to liver and the bronchograms appear crowded and parallel. Moreover in pneumonia air bronchograms can have intrinsic dynamic centrifugal movements due to breathing. The finding of dynamic air bronchogram on LUS attests bronchial patency and rules out atelectasis [26]. For purposes of analysis, subcentimeter bacterial pneumonia was defined as focal lung consolidations with air bronchograms with a size of less than one centimeter. According to literature [21–23], small subpleural consolidations with no air bronchograms (typically < 0.5 cm) with associated pleural line abnormalities, single or confluent B lines were considered associated with bronchiolitis or viral pneumonia (Fig. 1).

### CXR examination

In supine position, infants were acquired a posterior–anterior CXR. Lateral radiographs were not obtained to decrease radiation exposure, according to the British Thoracic Society Guidelines recommendations [10]. The radiologist was blind to the infants’ clinical and laboratory data. Bacterial pneumonia on CXR was defined as “consolidation”, “infiltrate”, or “pneumonia” identified by the pediatric radiology. Viral infections on CXR was diagnosed on the attending pediatric radiologist reading for “likely viral infiltrates”, “peri-bronchial infiltrates”, “peri-bronchial cuffing”, “peri-bronchial thickening”, or “increased interstitial markings” [21].

### Statistical analysis

Assuming a proportion of 22% of abnormal CXR inconsistent with simple bronchiolitis, comparable to data reported by Mahabee-Gittens [27], 87 patients were estimated to be enrolled to provide more than 90% power with a two-sided alpha level of 0.05.

We compared the clinical characteristics of patients with simple bronchiolitis with those of bronchiolitis and bacterial pneumonia using the chi-square test to compare proportions, the two-sample t test to compare average values, and the Mann–Whitney test to compare the median values between the groups.

We calculated sensitivity, specificity, positive predictive value (PPV) and negative predictive value (NPV) (95% confidence intervals [CIs]) of LUS and CXR in diagnosing bacterial pneumonia to assess tests performance [28]. The correlation between LUS and CXR results and between LUS changes and clinical/laboratory data was assessed through Spearman’s test. We considered the strength of the correlation as very weak (0.0–0.19), weak (0.20–0.39), moderate (0.40–0.59), strong (0.60–0.79) or very strong (0.80–1.0). Kappa statistics were calculated to examine the agreement between the pediatric and radiologist sonologist interpretations for a positive LUS. Similarly, Kappa statistics were calculated to assess the agreement between two radiologists’ interpretations for a positive CXR. We categorized the strength of agreement measured by the Kappa statistic as poor (< 0.0), slight (0.0–0.2), fair (0.2–0.4), moderate (0.4–0.6), substantial (0.6–0.8) or almost perfect (0.8–1.0) [29].

Data were analysed using the STATA version 10.0 software package. For all the analyses, significance was accepted at *p* < 0.05.

## Results

### Patients characteristics

A total of 87 patients (mean age 5.7 months ±5.2, 44 females) were enrolled in the study. The reasons for performing CXR were: fever > 38.5 °C or > 38 °C for 2 or more days in 20 patients, persistent SatO2 < 92% in 27 cases, asymmetric breath sounds on auscultation in 33 patients, WBC > 15,000/mmc in 25 cases, CRP > 4 mg/dl in 14 cases and septic appearance in 2 cases. A final diagnosis of concomitant bacterial pneumonia was done in 25/87 patients with bronchiolitis.

Table 1 summarizes the characteristics of patients with simple bronchiolitis and those with concomitant bacterial pneumonia. Children with pneumonia had significantly lower SatO2 (p 0.001) and higher CRP levels (p 0.023) and they required oxygen supplementation to maintain SatO2 ≥ 92% in a higher percentage (*p* < 0.0001) and for longer time (p < 0.0001) compared to the patients with simple bronchiolitis. Moreover the length of stay in hospital was longer in children with concomitant pneumonia compared to those with simple bronchiolitis (*p* 0.002).

**Table 1:** Demographic, clinical and laboratory data of patients with uncomplicated bronchiolitis and those with concomitant bacterial pneumonia

	Uncomplicated bronchiolitis (*n* = 62)	Bronchiolitis with concomitant bacterial pneumonia (*n* = 25)	*p* value
Months of age - mean (SD)	5.77 (5.49)	5.72(4.62)	0.965
Females n° (%)	29 (47)	15 (60)	0.264
Temperature °C, mean (SD)	37.9 (0.9)	38.2 (1.0)	0.107
Oxygen saturation %, mean (SD)	94.8 (3.5)	91.5 (4.4)	0.001
WBC count mmc, mean (SD)	12,278 (4815)	13,880 (5075)	0.170
Neutrophil count %, mean (SD)	43.7 (16.6)	49.3 (17.7)	0.169
Lymphocytes count %, mean (SD)	44.3 (15.9)	37.9 (13.5)	0.083
CRP mg/dl – mean (SD)	1.60 (2.44)	3.60 (3.89)	0.023
Bronchiolitis severity ^a^, n° (%)			0.029
Mild	13 (21)	0	
Moderate	29 (47)	12 (48)	
Severe	20 (32)	13 (52)	
Oxygen supplementation, n° (%)	26 (42)	21 (84)	< 0.0001
Hours of oxygen supplementation, mean (SD)	33.0 (42.5)	94.9 (71.4)	< 0.0001
Days of hospital stay, mean (SD)	4.8 (2.6)	7.1 (3.9)	0.002

*WBC* White Blood Cell, *CRP* C-Reactive Protein, *SD* Standard Deviation

^a^according to the Italian inter-society consensus document on bronchiolitis [50]

### LUS and CXR findings

Of the 25 patients with bacterial pneumonia, CXR was positive for parenchymal consolidation consistent with pneumonia in 24 cases. CXR showed false-positive findings in 8 children. In the only patient with a false-negative CXR, LUS showed a subcentimeter pneumonia in the posterior basal retrocardiac region of the left lung.

LUS was able to identify all the cases of bronchiolitis with concomitant bacterial pneumonia, of which 5/25 were subcentimetric pneumonia. According to literature [30], the majority of patients with pneumonia (16/25, 64%) had a sonographic consolidation in the posterior lung zones. In 6 patients LUS was able to identify 2 concomitant consolidations associated with bronchograms, thus the total number of ultrasound consolidations consistent with pneumonia was 31, of which 21 (67.8%) were in the posterior lung zones. LUS showed false-positive findings in 10 cases, all but one consisting in subcentimetric pneumonia. In the only patient with a false-positive consolidation > 1 cm on ultrasound, the final diagnosis was RSV pneumonia.

Table 2 summarizes the comparison of the CXR and LUS results in the diagnosis of bacterial pneumonia in our patients.

**Table 2:** Comparison of CXR and LUS findings. **A** Comparison of CXR and LUS results, including all consolidation size in the LUS positive findings; **B** Comparison of CXR and LUS results, including only consolidation size > 1 cm in the LUS positive findings

	Uncomplicated bronchiolitis			Bronchiolitis with concomitant bacterial pneumonia		
	CXR negative	CXR positive	Total	CXR negative	CXR positive	Total
**A**						
LUS negative	46	6	52	0	0	0
LUS positive	8	2	10	1	24	25
Total	54	8	62	1	24	25
**B**						
LUS negative	53	8	61	1	4	5
LUS positive	1	0	1	0	20	20
Total	54	8	62	1	24	25

**Fig. 2:**
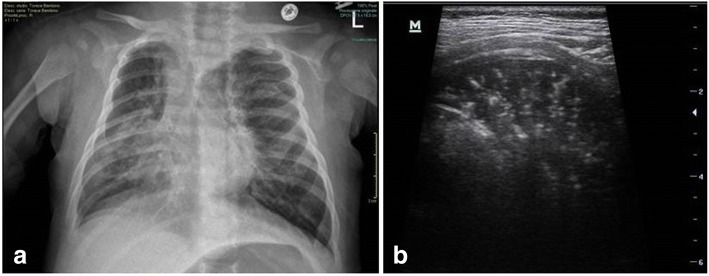
Comparison of CXR and LUS in a patient with bronchiolitis complicated by pneumonia in the right lung. **a** CXR showed a right lung consolidation consistent with pneumonia, associated with hyperinflation and a mediastinal herniation of the left lung. **b** LUS revealed a large hypoechoic consolidated area with sonographic air bronchograms with branching pattern, compatible with pneumonia

**Fig. 3:**
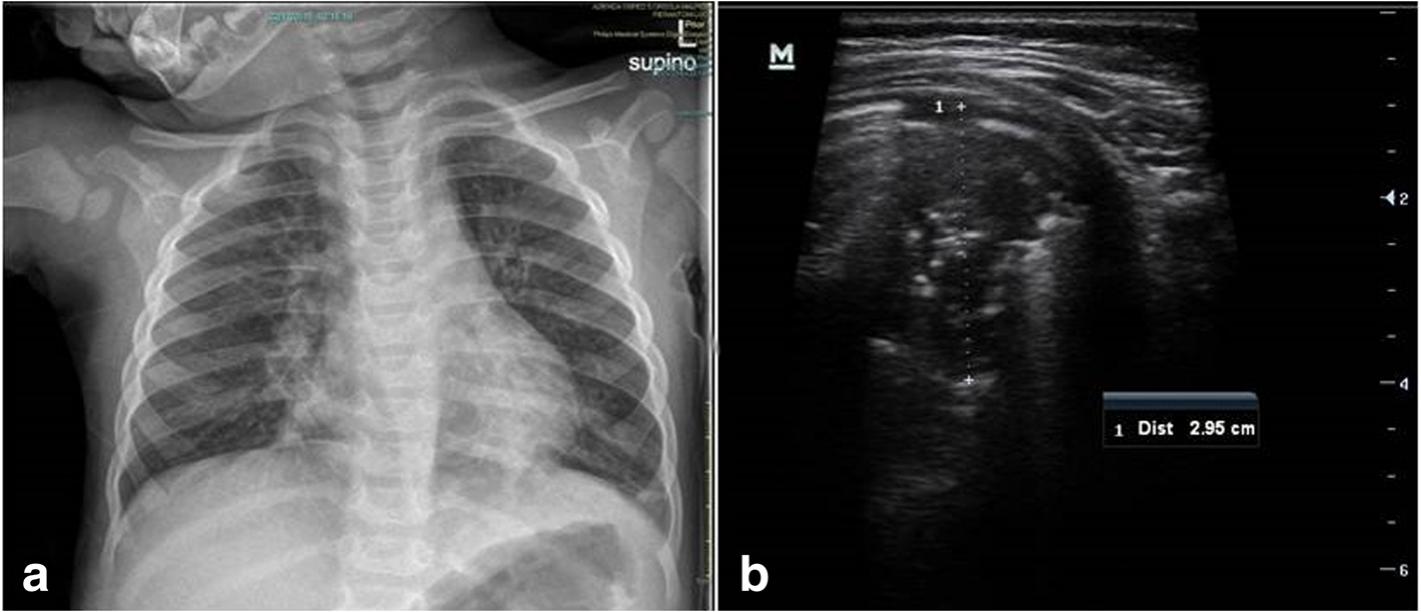
Comparison of CXR and LUS in a patient with bronchiolitis complicated by pneumonia in the left lung. **a** CXR demonstrated a basal left consolidation suggestive of pneumonia and bilateral peri-bronchial thickening. **b** Ultrasound showed a consolidation with air bronchograms in the posterior region of the left lung

Figure 2 and Fig. 3 show the CXR and LUS findings in two patients with a final diagnosis of bronchiolitis complicated by bacterial pneumonia.

**Fig. 4:**
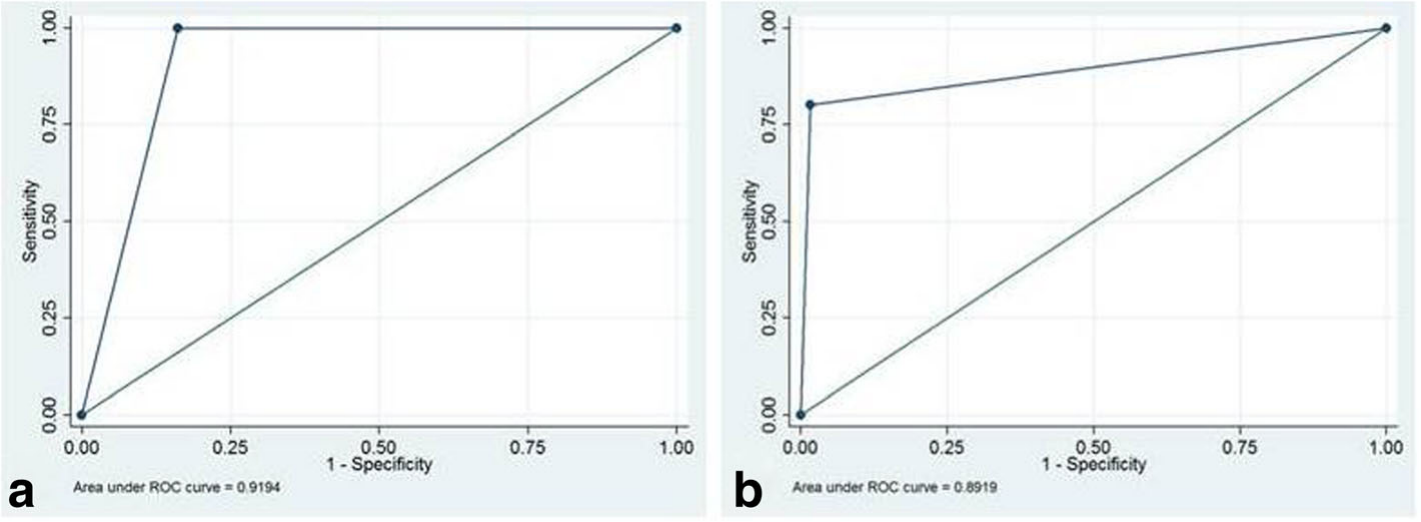
Receiver Operating Characteristic (ROC) curve for LUS for the diagnosis of bacterial pneumonia in children with bronchiolitis. **a** The ROC curve when all the consolidations associated with bronchogram were considered consistent with bacterial pneumonia. **b** The ROC curve when only ultrasound consolidations size > 1 cm were considered consistent with bacterial pneumonia

CXR showed a sensitivity of 96% (95% CI 88.8–98.8%) and specificity of 87.1% (95% CI 77.8–93.0%) in identifying children with bronchiolitis affected by a concomitant bacterial pneumonia, with a PPV of 75% (95% CI 64.4–83.4%) and a NPV of 98.2% (91.9–99.8%). LUS had a sensitivity of 100% (95% CI 94.7–99.9%) and a specificity of 83.9% (95% CI 74.1–90.6%); the PPV and NNV were 71.4% (95% CI 60.6–80.4%) and 100% (95% CI 94.7–99.9%) respectively. The area under the receiver operating characteristic (ROC) curve was 0.92 (Fig. 4a).

When only ultrasound consolidation size > 1 cm was considered consistent with pneumonia, LUS sensitivity was 80.0% (95% CI 69.8–87.5%) and specificity 98.4% (95% CI 92.2–99.8%), with a PPV and NNV of 95.2% (95% CI 87.8–98.4%) and 92.4% (95% CI 84.2–96.4%) respectively. The area under the ROC curve was 0.89, indicating very good discrimination (Fig. 4b).

Spearman’s rho test showed a strong correlation between CXR and LUS in diagnosing bacterial pneumonia (rs 0.638, *p* < 0.0001). When only consolidation size > 1 cm was considered positive for pneumonia, the correlation became stronger (rs 0.684, p < 0.0001). The Spearman test was used also to assess the relationship between LUS findings (consolidations with bronchograms) and clinical/laboratory data (fever> 38 °C, SatO2 < 92%, WBC > 15,000/mmc, CRP > 4 mg/dl). No strong correlation was founded. A weak positive correlation emerged between positive LUS and SatO2 < 92% (rs 0.338, p 0.001), CRP > 4 mg/dl (rs 0.248, p 0.021) and TC > 38 °C (rs 0.220, p 0.040) when all consolidations with bronchograms were included in the LUS positive findings. The positive correlation was confirmed between LUS findings and SatO2 < 92% (0.359, p 0.001) and CRP > 4 mg/dl (rs 0.291, p 0.006) when considering only ultrasound consolidation size > 1 cm.

### LUS and CXR inter-observer agreement

Cohen’s kappa between pediatrician and radiologist sonologists in the first 30 patients showed an almost perfect agreement in diagnosing bacterial pneumonia by LUS (K 0.93). Cohen’s kappa between two expert radiologists concerning the interpretation for a positive CXR in the same patients revealed a substantial, but less strong agreement (K 0.74). Regarding the characteristics of this patient group, 7/30 cases (23%) had a final diagnosis of bronchiolitis complicated by bacterial pneumonia. In all these patients LUS examination showed a consolidation with air bronchograms > 1 cm. In 4/23 patients with a final diagnosis of uncomplicated bronchiolitis, LUS showed a subcentimetric consolidation. CXR identified all the patients with bronchiolitis and concomitant pneumonia, while it showed a false-positive findings in 2/23 cases with uncomplicated bronchiolitis. We compared the image spectrum of CXR and LUS in these 30 patients with the spectrum of findings in the whole population, and we found no statistically significant differences (see Table 3). These data support the relevance of our results which referred to the first 30 patients enrolled but potentially reflect the interobserver agreement of LUS and CXR in the whole population.

**Table 3:** Comparison of the CXR and LUS findings between the exploratory population group and the whole study population

	Exploratory population group (30 pts)	Whole study population (87 pts)	*P* value
CXR			
Normal, n° (%)	13 (43,3%)	32 (36,8%)	0,763
Pneumonia, n° (%)	9 (30,0%)	32 (36,8%)	
Viral infiltrates, n° (%)	8 (26,7%)	23 (26,4%)	
LUS			
All consolidations with bronchograms, n° (%)	11 (36,7%)	35 (40,2%)	0,730
Consolidations > 1 cm with bronchograms, n° (%)	7 (23,3%)	21 (24,1%)	0,929
Consolidations without bronchograms, n° (%)	19 (63,3%)	66 (75,9%)	0,184

## Discussion

Bronchiolitis is the most common viral lower respiratory tract infection that affects children younger than 2 years [1]. The diagnosis is clinical and guidelines currently do not recommend the routine use of CXR [4]. However, CXR is still performed in a high percentage of cases, mainly to diagnose or rule out bacterial pneumonia requiring antibiotic treatment. The prevalence of pulmonary bacterial co-infection in bronchiolitis varies widely on literature, ranging from 9.7% [31] to 42% in severe cases admitted to ICU [32]. These variations may be due to several factors, including the age of children, the severity of bronchiolitis, the differences of assistance setting, the percentage of patients who underwent CXR, and the criteria to define CXR abnormal.

Our study investigated the reliability of LUS in discriminating children with uncomplicated bronchiolitis from those with concomitant bacterial pneumonia.

We observed a high rate of bronchiolitis complicated by bacterial pneumonia in our study (29%). This may depend on the highly selected setting of our patients, as we included only hospitalized children with bronchiolitis who underwent CXR because of the suspicion of concomitant bacterial pneumonia. The diagnosis of pneumonia was corroborated by the lower oxygen saturation (p 0.001) and higher CR*P* values (p 0.023) of these patients compared to those with uncomplicated bronchiolitis, together with their prompt response to antibiotic therapy.

In our study we found a strong correlation between CXR and LUS in diagnosing bacterial pneumonia (rs 0.64). Many recent studies reported similar findings, showing substantial agreement between the two techniques with kappa values of 0.64–0.89 [33, 34]. Discordant results may be partially due to the superior sensitivity of LUS to detect subcentimetric consolidations [16]. According to this, when only consolidation size > 1 cm was considered positive for pneumonia, the correlation in our study grew further (rs 0.68).

The good ability of LUS to diagnose pediatric pneumonia has been previously reported in literature [16, 20, 35] but none of these studies focused on the setting of children with acute bronchiolitis. Consolidation has to extend to the pleural surface to be visualized by ultrasound and the supraclavicular region and/or the area covered by the scapula can be difficult to explore by LUS. However, studies on adults shows that pneumonia reach the pleura in 92% of hospitalized patients [36] and up to 98% in the critically ill [37]. A recent meta-analysis done on pediatric patients with suspected pneumonia [18] revealed a higher sensitivity (96%, 95% CI 94–97%) but lower specificity (93%, 95% CI 90–96%) of LUS compared with adults data [38]. The higher sensitivity may depend on the smaller thorax size and the thinner chest wall of children that lead to better visualization of the lung parenchyma by LUS [36]. The lower specificity may be a result of non infiltrative processes including atelectasis, that are common in pediatric diseases as asthma or bronchiolitis and can be misinterpreted as pneumonia by ultrasound when the size of consolidation is small [18].

According to these results, in our series we found a higher sensitivity of LUS compared to CXR (100% vs 96%) with a slightly lower specificity (84% vs 87%) when all sonographic consolidations with bronchogram were considered consistent with bacterial pneumonia. When only > 1 cm consolidations were considered positive, the specificity of LUS increased from 83.9 to 98.4%, raising a question about the diagnostic value of sonographic subcentimetric pneumonia.

These findings were consistent to those previously reported in literature [16, 20, 35] that highlighted the uncertain pathological relevance of subcentimeter consolidations. LUS is generally useful for differentiating pneumonia from atelectasis caused by bronchial block to air entry [26]. In pneumonia the bronchogram appears like a branching echogenic structure and can have intrinsic dynamic centrifugal movements due to breathing (“dynamic air bronchogram”) proving bronchial patency and ruling out atelectasis. On the contrary, the bronchogram in atelectasis has a parallel course and it is typically a “static air bronchogram” due to the absence of airflow secondary to the occluded airway [26]. However the air bronchogram and its characteristics (parallel vs arborized, static vs dynamic) can be difficult to detect in subcentimeter lung consolidations. Subpleural consolidations - commonly present in bronchiolitis - can be misinterpreted as subcentimeter pneumonia by ultrasound. According to this, in our study LUS showed false-positive findings in 10 children, all but one consisting in subcentimeter pneumonia. LUS is without any radiation exposure and it requires short examination time (2–8 min) [16] and lower cost compared to CXR [20] as it can be performed by the pediatrician during the daily ward round with immediate bedside availability of results. As a result, we suggest that patients with subcentimeter consolidations should undergo ultrasound follow up before starting antibiotic therapy with the aim of identifying those who will achieve a spontaneous resolution. Moreover we evaluated the correlation between positive LUS (consolidations with bronchograms) and clinical/laboratory data (fever > 38 °C, SatO2 < 92%, WBC > 15,000/mmc, CRP > 4 mg/dl). No strong correlation was founded. This may be due to the fact that no clinical manifestations nor laboratory markers are able to clearly differentiate bacterial from viral disease and predict severity of pediatric pneumonia [39]. However a weak positive correlation emerged between positive LUS and SatO2 < 92%, CRP > 4 mg/dl and TC > 38 °C when all consolidations with bronchograms were included in the LUS positive findings. The positive correlation was confirmed between LUS findings and both SatO2 < 92% and CRP > 4 mg/dl when considering only ultrasound consolidation size > 1 cm. If our results will be further confirmed in a larger populations in multicenter studies, a clinical and laboratory picture consistent with pneumonia, associated with positive LUS findings would exclude the need to perform a CXR.

To examine the effect of experience on LUS accuracy, we evaluated the interobserver agreement between a trained pediatrician and a pediatric radiologist sonologists in the first 30 patients enrolled in the study. We found an excellent interobserver agreement with a kappa value of 0.93, according to previous studies (kappa 0.55–0.93) [16, 20, 40]. The distinctive feature of our study is that both users independently performed the ultrasounds, and this is important considering that LUS is an operator-dependent technique and incomplete chest exploration may cause diagnostic pitfalls. The emerged almost perfect interrater reliability (IRR) between novice and expert users supports that LUS is a basic easy-to-learn sonographic technique, as affirmed by the International Liaison Committee on Lung Ultrasound [41].

Moreover we calculated the interobserver agreement of CXR between two pediatric radiologists on the same set of 30 patients to compare the reliability of CXR with LUS. We found a high IRR for CXR in detecting consolidation but lower than LUS (kappa 0.74 versus 0.93). Only one study did a similar comparison: 50 of the LUS and CXR images were read by 4 radiologists to calculate the interobserver agreement in diagnosing pediatric pneumonia, resulting in a poor IRR for CXR and moderate for LUS (kappa 0.36 versus 0.55) [39]. Similarly, fair to moderate IRR for the interpretation of CXR for pediatric pneumonia is frequently reported in literature [42, 43], even if it varies considerably depending on level of prior training of reporters [44, 45]. In comparison with previous studies, the interobserver agreement of CXR in our study was unexpectedly high. This may have been because of the high experience level of our attending pediatric radiologists.

Our study had some limitations. First, this was a single center study with a relatively small numbers of children. Therefore, more studies with a larger sample size are required to confirm our data. The second limitation of the study is the lack of a true diagnostic reference standard due to ethical reasons. This is a common limitation in all studies assessing the diagnostic performance of LUS compared to CXR for the diagnosis of pneumonia in children. CT is normally considered the ideal gold standard for pneumonia, but it cannot be routinely used in children due to intensive exposure to radiations, availability and high cost. CXR is widely considered a crucial step in the diagnosis of pneumonia, nevertheless it is not 100% sensitive nor specific, and variation exists in intra- and interobserver agreement among radiologists [46]. The limitations of CXR in the diagnosis of pneumonia are more evident in patients with bronchiolitis, because radiographic appearance of infiltrate is similar to atelectasis [9] and CXR cannot reliably distinguish viral from bacterial pneumonia [10, 47]. Thus, according to previous studies [17, 48, 49], our diagnostic gold standard was the ex-post diagnosis of pneumonia made by an experienced pediatrician blinded to LUS findings, on the basis of clinical presentation, laboratory tests and CXR.

Despite these limitations, this study found useful results that support the reliability of LUS in evaluating pneumonia also in children affected by bronchiolitis, providing arguments for reducing CXR achievement.

## Conclusions

This study shows the good accuracy of LUS in diagnosing bacterial pneumonia in children with clinical bronchiolitis. When including only consolidation size > 1 cm, specificity of LUS was higher than CXR (98.4% versus 87.1%). These data suggest that a positive lung ultrasound > 1 cm may avoid the need to perform CXR in these patients. If our results will be confirmed by further studies on larger populations, the routine use of LUS for children with bronchiolitis and suspected bacterial pneumonia could reduce the number of CXR performed, decreasing the exposure to ionizing radiations and the medical costs. Regarding children with subcentimeter consolidations on LUS, we suggest careful clinical and ultrasound follow-up to discriminate patients who will need antibiotic treatment from those who will achieve a spontaneous resolution. In this sense a watchful waiting approach can be adopted for improved antibiotic stewardship. Moreover, the good inter-observer agreement between the pediatric clinician and the radiologist highlights that LUS is a technique easy to learn, and it can be potentially performed by the clinician in every setting with immediate bedside availability of results.

In summary, LUS may represent a value supplemental tool in the diagnostic work-up of bronchiolitis when a concomitant pneumonia is suspected. Further studies are required to validate the diagnostic accuracy of LUS on larger population and to evaluate the impact of LUS on antibiotic use and stewardship in children with complicated bronchiolitis.

## Abbreviations

CI: Confidence interval
CRP: C-reactive protein
CT: Computed tomography
CXR: Chest x-ray
ICU: Intensive care unit
LUS: Lung ultrasound
ROC: Receiver operating characteristic
RSV: Respiratory syncytial virus
SatO2: Oxygen saturation
WBC: White blood cells
